# Supplementary material for: Splice-Junction-Based Mapping of Alternative Isoforms in the Human Proteome
Source: Cell Rep. Author manuscript; Available in PMC 2020 Jan 15. (PMC6961840; doi:10.1016/j.celrep.2019.11.026)
Supplement: 3 [file NIHMS1546469-supplement-3.zip › DF2/PXD009737/TestisGluC-203-Q9UPN3-ITAPDSQGKTDLTE.pdf]

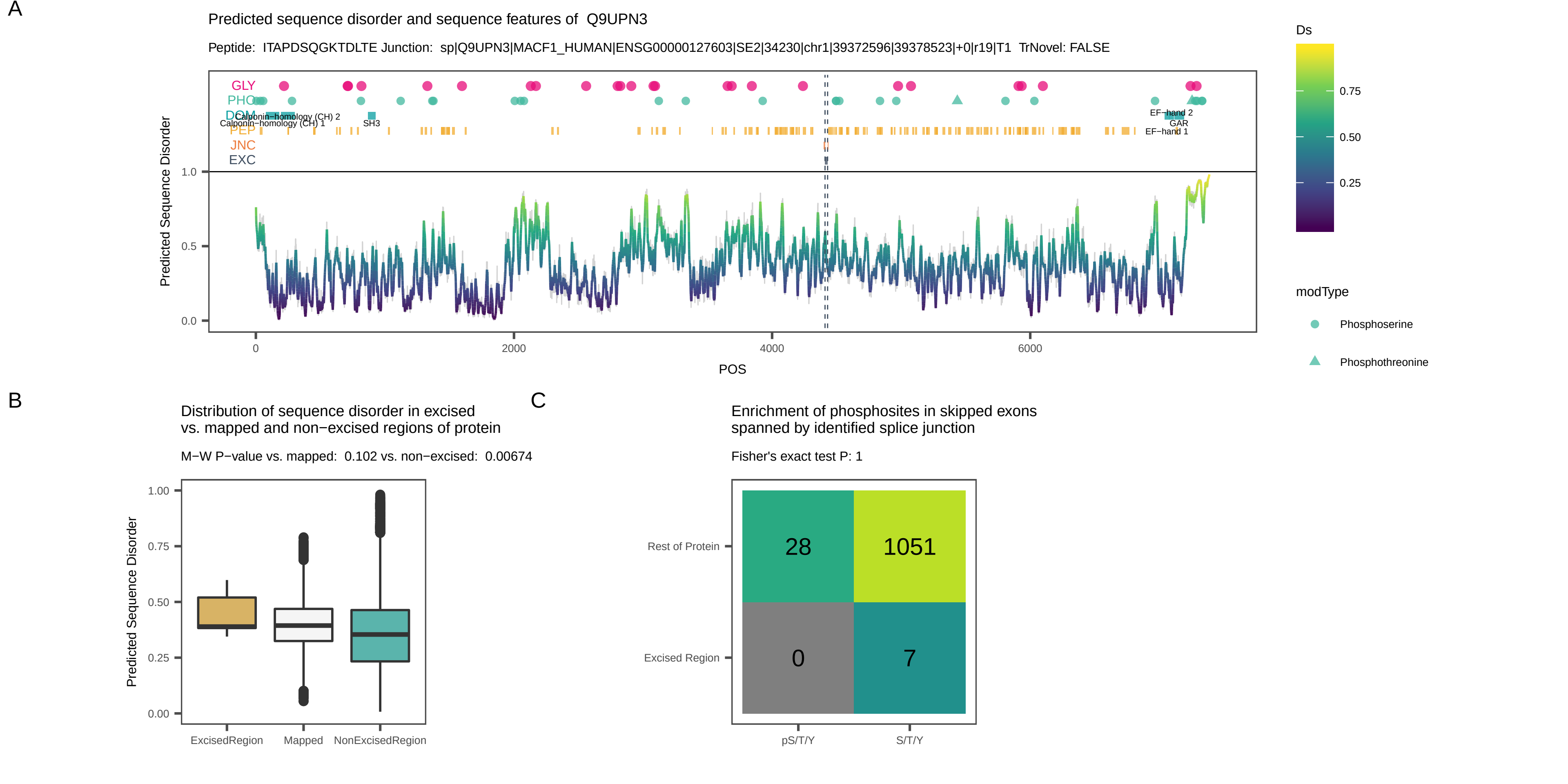
A
Predicted sequence disorder and sequence features of Q9UPN3
Peptide: ITAPDSQGKTDLTE Junction: sp|Q9UPN3|MACF1_HUMAN|ENSG00000127603|SE2|34230|chr1|39372596|39378523|+0|r19|T1 TrNovel: FALSE
GLY
PHO
DOM
PEP
JNC
EXC
Calponin−homology (CH) 2
Calponin−homology (CH) 1
SH3
EF−hand 2
GAR
EF−hand 1
Predicted Sequence Disorder
1.0
0.5
0.0
0
2000
4000
6000
POS
Ds
0.75
0.50
0.25
modType
Phosphoserine
Phosphothreonine
B
Distribution of sequence disorder in excised vs. mapped and non−excised regions of protein
M−W P−value vs. mapped: 0.102 vs. non−excised: 0.00674
Predicted Sequence Disorder
1.00
0.75
0.50
0.25
0.00
ExcisedRegion
Mapped
NonExcisedRegion
C
Enrichment of phosphosites in skipped exons spanned by identified splice junction
Fisher's exact test P: 1
Rest of Protein
Excised Region
28
1051
0
7
pS/T/Y
S/T/Y
